# Gaming disorder prevention among adolescents in Japan: Challenges and future directions

**DOI:** 10.1002/pcn5.70424

**Published:** 2026-10-14

**Authors:** Kai Kobayashi, Tsubasa Sasaki, Eiji Shimizu

**Affiliations:** ^1^ Department of Cognitive Behavioral Physiology, Graduate School of Medicine Chiba University Chiba Japan; ^2^ Research Center for Child Mental Development Chiba University Chiba Japan; ^3^ Center for Preventive Medical Sciences Chiba University Chiba Japan

Gaming disorder (GD) is characterized by impaired control over gaming, increasing priority given to gaming over daily activities, and continuation of gaming despite negative consequences.^1^ In Japan, the prevalence of GD has been estimated at 6.2% among adolescents aged 10–14 years and 7.6% among those aged 15–19 years.^2^ Because of its prevalence among adolescents, prevention of GD is urgently needed. This article reviews domestic and international preventive policies and initiatives and discusses GD prevention tailored to Japanese adolescents. Given the limited research on GD prevention, this letter focuses on GD while considering prevention studies on related concepts, including problematic internet use, internet addiction, and internet use disorder.

At the policy level, Kagawa Prefecture in Japan implemented a top‐down ordinance in 2020 to restrict adolescents' gaming time.^3^ Although the local government reported a slight numerical decline in gaming dependence tendencies, its statistical significance was not assessed. Therefore, it is difficult to determine the policy's effect definitively or to evaluate its overall merits. Furthermore, gamers may view restrictive measures unfavorably. Stevens et al.^4^ reported that gamers supported primary prevention strategies, including educational guidelines and self‐monitoring tools and apps. Participants reported the highest disapproval rating for in‐game shutdown features (65%). Regarding prevention policy in Europe, Lopez‐Fernandez and Kuss^5^ reviewed European policy options for preventing internet addiction problems and classified them into four categories: (1) no action, (2) responsible internet use—support professionals, (3) educate on health behaviors—support users, and (4) context of online users—support communities. In particular, supporting professionals involves disseminating research‐based information and providing self‐screening tools and other tests to professionals such as clinicians and teachers. Regarding support for users, the review highlighted the promotion of healthy internet use, with preventive actions including encouraging alternative entertainment behaviors, detecting the risk of comorbidities or other problems and addressing them with professional support and the support of significant others (e.g., caregivers for adolescents), and assessing other internet use‐related addictions when problematic internet use is identified.

At the intervention level, several initiatives relevant to GD prevention have been reported. In Japan, Tsuruta^6^ reported reductions in internet addiction tendencies and gaming time following a four‐year educational program involving 120 adolescents. Moreover, Shirasaka and Tsuneta^7^ conducted harm‐reduction‐based awareness‐raising activities through educational materials and lectures. In Germany, Lindenberg et al.^8^ evaluated a cognitive behavioral therapy (CBT)‐based prevention program for adolescents at risk of GD and unspecified internet use disorder in a cluster randomized clinical trial. At 12 months, the intervention group demonstrated a significantly greater reduction in symptom severity than the control group (39.8% vs. 27.7%; Cohen's *d* = 0.67). However, the difference in the incidence of new diagnoses between the two groups was not statistically significant.

Evidence on their effectiveness remains limited: the Japanese reports provided limited comparative evidence,^6^, ^7^ and the German RCT did not find a statistically significant difference in the incidence of new diagnoses.^8^ Regarding implementation, Tsuruta^6^ noted the need for teacher collaboration and consideration of students' burden in repeated assessments.

A recent notification from the Ministry of Education, Culture, Sports, Science and Technology promotes ICT‐based health observations to identify changes in students' physical and psychological conditions and link students to appropriate support as part of suicide prevention.^9^ The notification also emphasizes helping students recognize psychological distress and seek help. In this broader policy context, a school‐based web stress‐check program for junior and senior high school students offers online access to brief CBT‐based psychoeducational and self‐care materials.^10^ This program illustrates one possible format for providing supplementary web‐based mental health content in schools. We propose providing brief psychoeducation for GD through supplementary materials accompanying ICT‐based health observations. Drawing on the teaching practice,^6^ harm‐reduction approach,^7^ and CBT techniques,^8^ the psychoeducation would aim to help students understand GD, reflect on their gaming in relation to daily life, learn self‐care strategies, and seek help when problems arise. Following the format of existing 5‐min CBT self‐care exercises,^10^ the psychoeducation would comprise a short series of similarly brief activities that students could revisit as needed.

In the long term, the goals of GD prevention are to reduce the number of adolescents who develop GD and promote early detection and early intervention for adolescents at risk. The feasibility and effectiveness of the proposed program require evaluation. Furthermore, an interdisciplinary approach involving healthcare, education, informatics, and the gaming industry may help develop GD prevention tailored to the Japanese context. Finally, GD prevention should not rely on behavioral restrictions but support adolescents in enjoying gaming in moderation and promote their well‐being.

## AUTHOR CONTRIBUTIONS

Kai Kobayashi conceptualized the study and wrote the manuscript. Kai Kobayashi and Tsubasa Sasaki performed the investigation. Tsubasa Sasaki curated the data. Eiji Shimizu supervised the project. All authors reviewed and approved the final version of the manuscript.

## CONFLICT OF INTEREST STATEMENT

The authors declare no conflicts of interest.

## ETHICS APPROVAL STATEMENT

Not applicable.

## PATIENT CONSENT STATEMENT

Not applicable.

## CLINICAL TRIAL REGISTRATION

Not applicable.

## Data Availability

Data sharing is not applicable to this article as no datasets were generated or analyzed during the current study.
